# Linked-read genome sequencing identifies biallelic pathogenic variants in *DONSON* as a novel cause of Meier-Gorlin syndrome

**DOI:** 10.1136/jmedgenet-2019-106396

**Published:** 2019-11-29

**Authors:** Karen M Knapp, Rosie Sullivan, Jennie Murray, Gregory Gimenez, Pamela Arn, Precilla D'Souza, Alper Gezdirici, William G Wilson, Andrew P Jackson, Carlos Ferreira, Louise S Bicknell

**Affiliations:** 1 Department of Pathology, Dunedin School of Medicine, University of Otago, Dunedin, New Zealand; 2 MRC HGU, Medical Research Council Human Genetics Unit, Institute of Genetics and Molecular Medicine, Edinburgh, UK; 3 Nemours Children's Clinic, Jacksonville, Florida, USA; 4 Office of the Clinical Director, National Human Genome Research Institute, Bethesda, Maryland, USA; 5 Department of Medical Genetics, Kanuni Sultan Suleyman Training and Research Hospital, Istanbul, Turkey; 6 Department of Pediatrics, University of Virginia School of Medicine, Charlottesville, Virginia, USA; 7 Medical Genomics and Metabolic Genetics Branch, National Human Genome Research Institute, Bethesda, Maryland, USA

**Keywords:** linked-read genome sequencing, meier-gorlin syndrome, *donson*, DNA replication, microcephaly

## Abstract

**Material:**

Linked-read whole genome sequencing (WGS) presents a new opportunity for cost-efficient singleton sequencing in place of traditional trio-based designs while generating informative-phased variants, effective for recessive disorders when parental DNA is unavailable.

**Methods:**

We have applied linked-read WGS to identify novel causes of Meier-Gorlin syndrome (MGORS), a condition recognised by short stature, microtia and patella hypo/aplasia. There are eight genes associated with MGORS to date, all encoding essential components involved in establishing and initiating DNA replication.

**Results:**

Our successful phasing of linked-read data led to the identification of biallelic rare variants in four individuals (24% of our cohort) in *DONSON*, a recently established DNA replication fork surveillance factor. The variants include five novel missense and one deep intronic variant. All were demonstrated to be deleterious to function; the missense variants all disrupted the nuclear localisation of DONSON, while the intronic variant created a novel splice site that generated an out-of-frame transcript with no residual canonical transcript produced.

**Conclusion:**

Variants in *DONSON* have previously been associated with extreme microcephaly, short stature and limb anomalies and perinatal lethal microcephaly-micromelia syndrome. Our novel genetic findings extend the complicated spectrum of phenotypes associated with *DONSON* variants and promote novel hypotheses for the role of DONSON in DNA replication. While our findings reiterate that MGORS is a disorder of DNA replication, the pathophysiology is obviously complex. This successful identification of a novel disease gene for MGORS highlights the utility of linked-read WGS as a successful technology to be considered in the genetic studies of recessive conditions.

## Introduction

DNA replication is a core cellular process for proliferation and organism growth. In addition to the core components structurally required for polymerase loading and activation, there are many accessory factors involved in regulation and surveillance, all of which are considered essential for organism development. Human genetic studies have identified hypomorphic pathogenic variants in these components in several developmental syndromes of restricted growth,^1^ with Meier-Gorlin syndrome (MGORS; MIM: 224690) the most closely linked to the initiation of DNA replication.

MGORS is clinically defined by a triad of features: short stature, microtia and patellae hypo/aplasia. It is common for MGORS individuals to have microcephaly and in fact the short stature and microcephaly is often proportionate.^2^ The severity of growth restriction can be variable, ranging from severe growth defects in keeping with other forms of primordial dwarfism such as microcephalic osteodysplastic primordial dwarfism (MOPD II) (MIM: 210720),^1 3 4^ through to normal growth parameters in some MGORS individuals.^5 6^ Facially, affected individuals have a recognisable phenotype with a thick vermillion of the lower lip, microstomia and micrognathia.^6^ Additional phenotypic features include skeletal and joint abnormalities, genital hypoplasia and absence of breast development in females.^6 7^

Gene discovery efforts have determined that MGORS, a normally autosomal recessive condition, is a disorder of DNA replication initiation. The first genes identified underlying MGORS were *ORC1*, *ORC4*, *ORC6*, *CDT1* and *CDC6,* 3 8 9 all encoding members of the prereplication complex (preRC), which acts to licence genomic origins of DNA replication. Pathogenic variants in these genes are hypomorphic loss-of-function alleles and cause reduced preRC formation on chromatin, impacting replication efficiency.^3^ Subsequent to this, biallelic variants in *MCM5*, encoding a subunit of the MCM2-7 helicase complex, were described in a single individual with MGORS,^10^ as well as de novo gain-of-function variants in *GMNN*,^11^ a negative regulator of CDT1. CDC45, an essential component of the preinitiation complex (preIC), the next step in the initiation pathway, has also been associated with MGORS and/or craniosynostosis.^12^ CDC45 interacts with GINS1-4 and the MCM helicase to form the CMG helicase, which once activated by these interactions, unwinds DNA to allow polymerase access and commencement of DNA replication.^13^ Together, these genetic findings strongly support the notion that MGORS is due to disruption of origin licencing and firing in the earliest steps of DNA replication. A delay in cell cycle progression likely underlies the growth retardation seen in these individuals^1 3^; however, the pathophysiological mechanism causing the microtia and patellar a/hypoplasia remains unclear.

Genome sequencing has supported the identification of novel Mendelian genes in a hypothesis-free manner, furthering biological and clinical insight into the pathophysiology of rare disorders. Here, we describe the use of linked-read Chromium genome sequencing technology to identify biallelic pathogenic variants *in trans*, without the need to sequence parents. Our genetic evidence, combined with functional studies demonstrating all variants are deleterious, indicates *DONSON* is a novel disease gene underlying MGORS. This further expands the phenotypic spectrum linked to DONSON disruption; pathogenic variants in *DONSON* cause both proportionate and disproportionate reduction in body and brain growth, as well as a wide variety of skeletal anomalies. Given all previous genes disrupted in MGORS are associated with early stages of DNA replication, our findings suggest DONSON may have yet unappreciated novel roles in DNA replication initiation.

## Materials and methods

### Ethics

Patient 4 was seen at the National Institutes of Health Clinical Center and enrolled in protocol 15-HG-0130, ‘Clinical and Genetic Evaluation of Individuals with Undiagnosed Disorders through the Undiagnosed Diseases Network’.^14–16^ All participants provided informed consent.

### Chromium genome sequencing and bioinformatics

DNA extraction was performed from whole blood using standard clinical laboratory protocols. Chromium genome sequencing was undertaken for samples P1–P3 at the Kinghorn Centre for Clinical Genomics, Garvan Institute of Medical Research (Sydney, Australia). Chromium genome libraries were prepared using a Chromium Genome Reagent Kit (v2 Chemistry) as per manufacturer’s instructions. The barcoded libraries were sequenced on an Illumina HiSeq X system with 150 bp paired-end reads, and the raw sequence data were converted to FastQ file format using Illumina’s bcl2fastq 2.16.0. The Long Ranger bioinformatics pipeline (V.2.1.6, 10X Genomics) was used to align reads against the human genome reference sequence (build GRCh37/hg19) and to generate a phased call-set of single nucleotide variants, insertion/deletions (indels) and large structural variants. Variants were annotated using snpEff, dbNFSP and with minor allele frequencies (MAF) from ExAC, gnomAD and 1000 Genomes.

Variants were first filtered based on a MAF <0.5%. Under a hypothesis of autosomal recessive inheritance, candidate disease genes (and their variants) were prioritised if they contained either homozygous variants or compound heterozygous variants within the same phase block as identified through Long Ranger. In addition, identified variants were prioritised based on functional impact, presence in disease databases and were parsed with a gene panel consisting of 291 genes with a known involvement in DNA replication (GO:0006260).

Trio exome sequencing was undertaken for sample P4 as described previously,^17^ and parallel bioinformatics were undertaken in-house from provided BAM files. HaplotypeCaller, as part of GATK v3 best practice guidelines, was used to call variants. These were then annotated and filtered as per above and parsed with the same gene panel.

Sanger sequencing was used to confirm the presence of variants in patient DNA (and parental DNA where available). *DONSON* sequencing primers are described in online supplementary material table S1. Variant nomenclature is based on RefSeq: NM_017613.3.

10.1136/jmedgenet-2019-106396.supp1 Supplementary data

### Protein alignment

Amino acid sequences for DONSON from different species were aligned using the default parameters of Clustal Omega and coloured based on percentage identity using Jalview. Refseq sequences were used where possible: *Homo sapiens* (NP_060083.1), *Macaca mulatta* (XP_001091031.1), *Canis familiaris* (XP_003640126.1), *Bos taurus* (NP_001076914.1), *Mus musculus* (NP_068366.1), *Rattus norvegicus* (NP_001008288.1), *Gas gallus* (XP_004934578.1), *Drosophila melanogaster* (NP_649531.1), *Anopheles gambiae* (XP_315901.4) and *Xenopus tropicalis* (NP_989384.1).

### Immunofluorescence

Plasmids expressing green fluorescent protein (GFP)-tagged DONSON were a kind gift from G Stewart, University of Birmingham. Site-directed mutagenesis was used to introduce the variants of interest into the pGFP-DONSON plasmid using Phusion Flash polymerase and then plasmids were verified by Sanger sequencing, with primers described in online supplementary material table S1. HeLa cells on coverslips at 80% confluence were transfected with plasmid DNA using Lipofectamine 2000 (Invitrogen). After 24 hours, cells were fixed and then permeabilised with methanol, then blocked with 7% FBS, 0.1% Triton X-100 in PBS and stained with DAPI. Slides were viewed using the Olympus BX53 upright microscope using CellSens Dimensions software. Five replicates were counted, with a total of approximately 1200 cells per plasmid. The proportion of cells with nuclear staining was compared with wild-type EGFP-DONSON for each construct, and p values were determined with a one-way analysis of variance, allowing for multiple comparisons.

### Minigene splicing assay

*DONSON* exons 3–5 along with flanking 5′ and 3′ intronic sequences (>200 bp) was PCR amplified from patient and control genomic DNA. Gateway cloning was used to insert PCR products into the pSpliceExpress vector^18^ (Addgene: #32485), which was a kind gift from Stefan Stamm. All constructs were verified by Sanger sequencing, with primers described in online supplementary material table S1.

HeLa cells were transiently transfected with plasmid DNA using Lipofectamine 2000 (Invitrogen). After 24 hours, RNA was extracted and purified using a Qiagen RNeasy Mini Kit (Qiagen), and then cDNA was synthesised using the SuperScript IV First-Strand Synthesis System (Thermo Fisher Scientific) with an oligo-dT primer. cDNA was PCR amplified using plasmid specific primers initially, and then using a combination of plasmid and plasmid/DONSON fusion primers to obtain sufficient amplification for gel extraction and Sanger sequencing. PCR products were visualised by gel electrophoresis, gel bands extracted using a GeneJET Gel Extraction kit (Thermo Fisher Scientific) and sequenced.

## Results

### Chromium-WGS reveals *DONSON* as a novel candidate disease gene for MGORS

To identify novel disease genes causing MGORS, we undertook genome sequencing of a cohort of 16 affected individuals. Given the autosomal recessive inheritance previously shown for MGORS, we took advantage of novel linked-read 10X Genomics Chromium genome sequencing technology to phase variants from Illumina genome sequencing to identify biallelic gene variants. This permitted phasing of variants even in cases where parental DNA was unavailable. Summary statistics from the Long Ranger pipeline showed an average genome coverage of 33.8X, an N50 phase block length of 1 924 048 and phasing of >98.6% of SNPs (online supplementary material table S2). Given the median gene length is 26 288 bp,^19^ the N50 phase block lengths provided confidence that variants within a single gene were able to be phased for the majority of genes in the genome. Prior to sequencing, we expected that phase block lengths would likely be smaller for P1, given the ancestral consanguinity present reducing the number of heterozygous SNPs, and therefore the ability to resolve informative haplotypes. Instead, we found that the underlying variability in input DNA molecule length contributed more heavily to the variability in phase block length (online supplementary material table S2).

We identified three individuals (P1–P3) harbouring clearly phased biallelic variants in *DONSON*. Subsequent trio exome sequencing of an additional MGORS patient (P4) also identified compound heterozygous *DONSON* variants, bringing the total number of MGORS individuals with variants in *DONSON* to four (table 1). These variants included five novel missense variants, one of which (p.Tyr270Cys) was recurrent in two individuals, as well as a deep intronic variant predicted to be a novel splicing variant. Segregation of all biallelic *DONSON* variants identified was confirmed by Sanger sequencing for families where parents were available. Given the rarity of MGORS and the genetic heterogeneity, finding four individuals with variants in the same gene (24% of the cohort) indicates *DONSON* is a relatively common MGORS disease gene.

**Table 1:** Variants identified in *DONSON* in individuals with Meier-Gorlin syndrome

Patient	Variant 1	gnomAD MAF	Variant 2	gnomAD MAF	Segregation
P1	c.631C>T, p.Arg211Cys	NA	c.631C>T, p.Arg211Cys	NA	Both parents heterozygous.
P2	c.494T>C, p.Phe165Ser	0.00001768	c.607–36G>A (splicing)	0.00005540	Mother heterozygous for c.706–36G>A, homozygous reference for c.494T>C, p.Phe165Ser.
P3	c.1634C>T, p.Pro545Leu	0.00001061	c.809A>G, p.Tyr270Cys	0.00001599	No parents available.
P4	c.670C>T, p.Pro224Ser	0.00006369	c.809A>G, p.Tyr270Cys	0.00001599	No parents available.

RefSeq:NM_017613.3.

As part of a larger collaboration, we had previously identified pathogenic variants in *DONSON* underlying severe disproportionate microcephaly and short stature, with limb anomalies (MISSLA; MIM #617604) in 29 individuals.^20^ All subjects had disproportionately severe microcephaly with mild short stature and a wide spectrum of other congenital anomalies. Skeletal abnormalities were common though variable. Three affected siblings from one family had previously been clinically diagnosed with a Fanconi anaemia-like disorder based on microcephaly and thumb dysplasia.^20 21^ A more recent MISSLA case report of German siblings, one of whom was also previously clinically diagnosed with Fanconi anaemia, described a similar phenotype.^22^ Separately, an ancestral intronic splicing pathogenic variant was found to cause microcephaly-micromelia syndrome (MIMIS; MIM #251230) segregating in a First Nations community.^23^ This syndrome is perinatal lethal with features of severe microcephaly, severe skeletal malformations of micromelia and digit anomalies, as well as characteristic craniofacial dysmorphologies.^23 24^ DONSON has been implicated in S phase and cell proliferation predominantly through study of the Drosophila orthologue *humpty dumpty*,^25 26^ with our detailed investigation revealing DONSON is a replication stress surveillance protein, which interacts with members of the replisome and is involved in ATR signalling as part of the DNA damage response.^20^ This described functional role in DNA replication, alongside our significant genetic evidence, strongly suggests *DONSON* is a novel disease gene associated with MGORS.

### Clinical characterisation of DONSON-MGORS individuals

**Figure 1:**
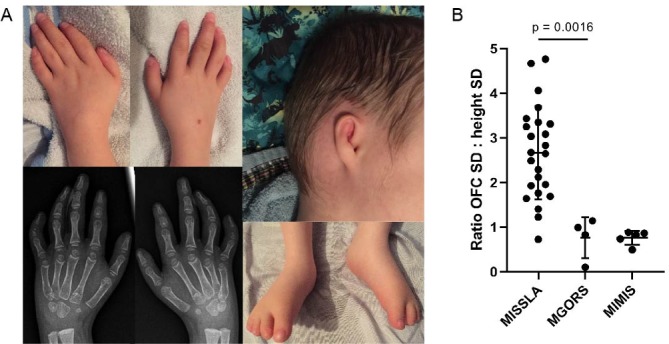
Clinical characteristics of DONSON-MGORS individuals. (A) Photographs of P4 showing tapering fingers, fifth finger clinodactyly caused by hypoplasia of the middle phalanx, small and simply formed ears and shortened toes with oedema evident. (B) Ratio of occupitofrontal circumference (OFC) to height SDs (z-scores) in MISSLA (taken from ref 15), DONSON-MGORS and MIMIS cohorts (taken from ref 17). Note that MISSLA and DONSON-MGORS measurements were taken at the most recent clinical exam, whereas MIMIS were taken at birth. While the most commonly associated syndrome with *DONSON* variants, MISSLA, shows a skewing of growth parameters due to the extreme disproportionate microcephaly, DONSON-MGORS shows proportionate reduction in size (unpaired t-test, p=0.0016). Separate consent for publication of images was provided by P4. MGORS, Meier-Gorlin syndrome; MIMIS, microcephaly-micromelia syndrome; MISSLA, microcephaly and short stature, with limb anomalies.

All individuals were clinically diagnosed with MGORS at the outset of this study (table 2). All DONSON-MGORS individuals had the characteristic features of short stature, microtia and absent patella. Three out of four individuals were proportionately small, with P3 having significant short stature but no microcephaly. Growth charts were only available for P4; these showed reduced growth parameters at birth followed by a normal growth velocity, in keeping with other MGORS individuals.^2^ Conductive hearing loss was reported in P1 and P4 and is presumably related to the microtia, as is observed in other individuals with MGORS.^6^ Photos were only available for P4, who displayed characteristic MGORS facies of downslanting palpebral fissures and full lips (not shown). His ears were very small and simply formed. He has tapered fingers with fifth finger clinodactyly and shortened toes with oedema in both feet (figure 1A).

**Table 2:** Clinical features of MGORS individuals with biallelic *DONSON* variants

Patient Country		Growth								Clinical features				
	Sex	Gestn (weeks)	Lgt cm (SD)	OFC cm (SD)	Weight kg (SD)	Recent exam (age):	Height cm (SD)	OFC cm (SD)	Weight kg (SD)	Development	Facial dysmorphism	Skeletal	Hearing	Other features of note
**P1** Turkey	M	40	na	na	1.5 (−5.28)	9 years 6 months	115 (−3.48)	48 (−3.97)	na	Delayed development,	Small dysplastic ears, high arched palate, micrognathia.	Absent patella.	Mild hearing loss.	Hypopigmentation.
**P2** USA	M	39	na	na	2.35 (−2.36)	7 years	98 (−4.62)	46.4 (−4.59)	13 (−5.46)	Delayed motor development (walking 3.5 years).	Prominent forehead, triangular face.	Bilateral absent patella.	na	Bilateral clubfeet, avascular necrosis of right hip.
**P3** USA	F	39	46 (−1.51)	34 (−0.11)	2.2 (−2.28)	29 years 10 months*	142 (−3.62)	55 (−0.37)	93 (+2.88)	Normal	Ear anomalies	Absent patella	na	Diabetes (possibly steroid induced), treated with GH as teenager, haemoptysis and deceased.
**P4** USA	M	32	37 (−1.97)	28 (−0.94)	1.08 (−1.85)	8 years 0 months	98.4 (−5.26)	47 (−4.35)	12.3 (−7.27)	Motor and speech delay, hypotonia and normal cognition	Microtia, submucosal cleft and bifid uvula.	Bilateral congenital knee dislocations, hyperextension and dislocation of both knees at birth and bilateral absent patella.	Conductive hearing loss (EAC stenosis).	Spontaneously resolved nystagmus, mild left optic nerve hypoplasia, bilateral inguinal hernia and bilateral single incomplete palmar creases.

Gestn (gestation), Lgt (length), OFC (occipital frontal circumference), SD (SD from the mean for age and sex). All birth parameter SDs calculated using Fenton growth charts,^29^ recent exam SDs calculated using the LMS growth calculator.

*Height and weight SDs based on 23 years of age, OFC SD based on 17 years of age.

EAC, external auditory canal; GH, growth hormone; MGORS, Meier-Gorlin syndrome.

A variety of skeletal anomalies have been previously associated with *DONSON* variants.^20 22 23^ While MGORS individuals can also have a variety of skeletal defects,^7^ these are not commonly reported. All four DONSON-MGORS subjects have the characteristic absent patella, but P2 also had bilateral clubfeet. P4 had winged clavicles, thin ribs, mild scoliosis and slender long bones. He has delayed bone age, and middle and distal phalanges were small (particularly bilateral middle phalanx of fifth fingers) or subluxed (particularly distal phalanges in left foot) (figure 1A).

DONSON-MGORS individuals display quite a different growth profile compared with the MISSLA cohort (table 2, figure 1B). In comparing the ratio of OFC (occupitofrontal circumference) to height z-scores (SD), where a proportionate reduction would equal 1, MISSLA individuals have an increased ratio (median=2.66), caused by severe disproportionate microcephaly with only mild short stature. In DONSON-MGORS individuals, the ratio is significantly different (p=0.0016) and is much closer to 1, reflecting the proportionate reduction in growth. In P3, the ratio is 0.11, where he has a more extreme short stature compared with microcephaly—the opposite to all MISSLA individuals. Using birth measurements,^23^ MIMIS individuals also have a ratio closer to 1 (median=0.83), where the limb micromelia has shortened the body length in combination with severe microcephaly.

We compared the common features of this DONSON-MGORS cohort with MISSLA and MIMIS associated with biallelic variants in *DONSON* (table 3). While there are certainly some overall commonalities, particularly in growth delay and with skeletal anomalies, there are also specific clinical signs associated with each group, which may be of clinical utility for differential diagnoses and for gaining insight into any genotype–phenotype correlations.

**Table 3:** Comparison of characteristic clinical features present across the DONSON: MISSLA–MIMIS–MGORS spectrum

Clinical features	MISSLA	MIMIS	DONSON-MGORS
**Growth**			
Microcephaly	++	++	+
Short stature	+	++	+
Proportionality in size	−	+*	+
**Skeletal**			
Craniosynostosis	−	+	−
Micromelia	+/-	++	−
Radial ray/thumb	+/-	++	−
Patellar a/hypoplasia	+/-	NA†	*
**Facies**			
Characteristic craniofacies	−	++	NA‡
Microtia	+	−	++
Low-set/abnormally rotated ears	+	+	++
**Other**			
Lethality	−	++	−

++=strong association (defining feature), +=common association, +/−=less common association, −=not associated.

*The proportionate reduction in size in MIMIS individuals is based on the micromelic lower limbs reducing height, rather than an overall growth restriction.

†The perinatal lethality prevented examination of the patella, which is a later ossifying bone.

‡Photos were not available for review in all cases.

MGORS, Meier-Gorlin syndrome; MIMIS, microcephaly-micromelia syndrome; MISSLA, microcephaly and short stature, with limb anomalies.

### Novel *DONSON* variants identified in MGORS individuals are deleterious to protein function

**Figure 2:**
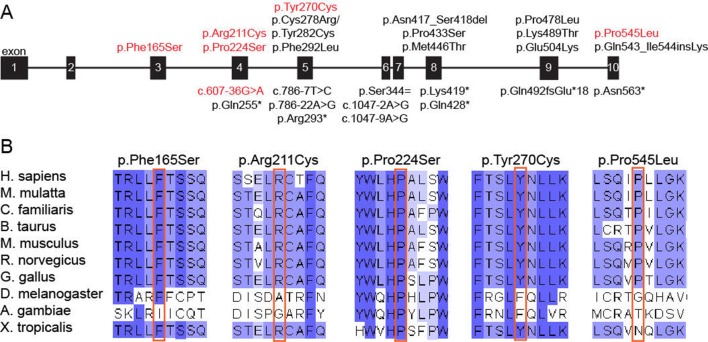
Molecular characteristics of DONSON-MGORS variants. (A) Schematic of the *DONSON* gene, with published pathogenic variants (black) and DONSON-MGORS variants (red) noted. Missense variants are listed above the gene cartoon, whereas splicing, truncating or loss-of-function variants are below. DONSON-MGORS are generally located closer to the N-terminus of the encoded protein, except for p.Pro545Leu, which is at the C-terminus. (B) Clustal alignment of DONSON from orthologous species. All residues at which substitutions occur are well conserved throughout evolution. While Pro545 does not demonstrate as strong conservation as the other residues, its position adjacent to a previously described variant provides additional evidence that such a substitution would be deleterious. MGORS, Meier-Gorlin syndrome.

**Figure 3:**
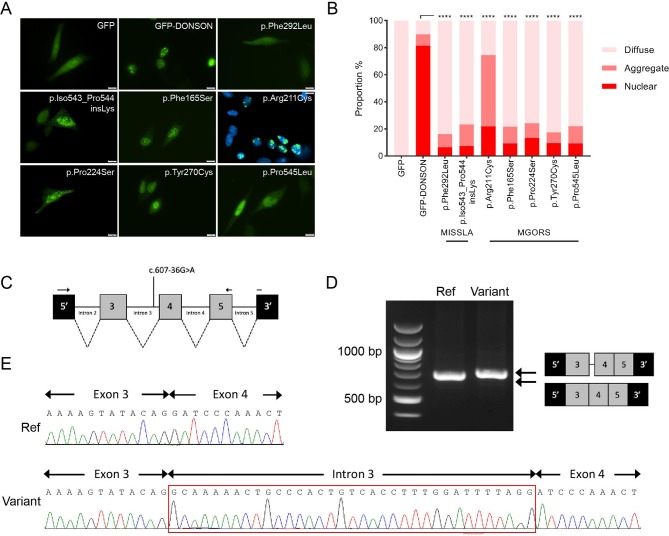
Functional analysis of novel DONSON-MGORS variants. (A) Representative images of DONSON subcellular localisation comparing GFP-DONSON to GFP-DONSON harbouring MGORS missense variants identified in this study (p.Phe165Ser, p.Arg211Cys, p.Pro224Ser, p.Tyr270Cys and p.Pro545Leu), as well as two previously identified pathogenic variants (p.Phe292Leu and p.Iso543_Pro544insLys). The p.Arg211Cys is co-stained with DAPI to highlight the nuclear aggregates present. Patient-identified variants all show diffuse signal throughout the nucleus and cytoplasm, compared with GFP-DONSON, which is only nuclear. (B) Quantification of DONSON subcellular localisation in figure part A (five biological replicates, n=1200 cells per plasmid, one-way ANOVA allowing for multiple comparisons). (C) Schematic of the *DONSON* gene included in the mini-gene assay, with the variant position and RT-PCR oligonucleotides indicated. (D) Agarose gel of the RT-PCR products from HeLa cells transfected with plasmids containing either the *DONSON* reference sequence (‘Ref’) or the *DONSON* c.607–36G>A variant (‘variant’). (E) Sanger sequencing of the RT-PCR products in figure part D, indicating a 34 base pair (bp) insertion into the transcript. ANOVA, analysis of variance; MGORS, Meier-Gorlin syndrome.

All variants identified in *DONSON* in this cohort have not been previously associated with disease. Of the five missense variants, four are found to be located closer to the N-terminus when compared with previously identified variants (figure 2A), and all affected amino acid residues are highly conserved across species (figure 2B). The remaining missense substitution (p.Pro545Leu), located towards the C-terminus, is less well conserved but lies adjacent to a site previously identified in MISSLA siblings (p.Gln543_Ile544insLys),^20^ suggesting this region of the protein requires structural fidelity. To examine the consequences of these *DONSON* missense variants on subcellular localisation, HeLa cells were transfected with a plasmid encoding either GFP-DONSON or GFP-DONSON containing the missense variants introduced by site-directed mutagenesis and then fixed 24 hours post-transfection. Fluorescence microscopy was used to assess subcellular localisation, and the percentages of GFP positive cells that contained either a solely nuclear GFP signal, protein aggregates or a diffuse pan-cellular GFP signal were quantified. In contrast to GFP-DONSON, which is targeted to the nucleus, the five DONSON-MGORS missense variants cause predominantly diffuse pan-cellular localisation (figure 3A), as has previously been shown for pathogenic MISSLA missense variants.^20^ Similar proportions of nuclear localisation were observed for the DONSON-MGORS and MISSLA variants compared with GFP-DONSON (p<0.0001). The exception was the p.Arg211Cys variant from P1, which had a significantly higher proportion of cells with aggregated DONSON protein that has not been observed for *DONSON* variants before, possibly suggesting that the variant causes protein misfolding. The structure of DONSON has not yet been solved, and given the predominance of missense variants identified here, understanding the structural consequences of the DONSON-MGORS missense variants could provide further insight.

P2 inherited a deep intronic variant in trans with a missense variant. The intronic variant identified (c.607–36G>A) lies beyond the proximity to exons where variants are usually associated with splice site alterations and in fact may not have been identified with an exome probe-based capture sequencing strategy. *In silico* splice site prediction using Alamut Visual (SOPHiA Genetics), which uses four different prediction algorithms, predicted a strong likelihood that this variant would create a new splice acceptor site 34 base pairs (bp) upstream of the canonical splice acceptor site for exon 4 (online supplementary material figure S1). Inclusion of these bases into the transcript would alter the reading frame and introduce a premature stop after 17 amino acids, likely causing nonsense-mediated decay of this transcript. To investigate whether this deep intronic variant altered canonical splicing, we used a minigene splicing assay as patient-derived RNA was not available. We introduced the genomic regions encoding *DONSON* exons 3–5 plus flanking 5′ and 3′ intronic sequences (≥200 bp) into the pSpliceExpress vector.^18^ Plasmids encoding either the reference or the c.607–36G>A variant sequence were transfected into HeLa cells, and the mRNA transcripts analysed by RT-PCR (figure 3C). PCR products were visualised by gel electrophoresis and, in accordance with *in silico* predictions, revealed differing gel bands for the reference and variant sequences (figure 3D). Sequencing of the RT-PCR products confirmed that the c.607–36G>A variant created a novel splice acceptor site, which resulted in the inclusion of a 34 bp sequence upstream of the known splice acceptor site for exon four as predicted (figure 3E). Notably, there was no canonical transcript produced from the c.607–36G>A-containing plasmid, suggesting this new splice site is strong enough to prevent splicing at the canonical exon–intron boundary. Given the shift in reading frame, this variant likely causes a null allele.

## Discussion

In this study, we have applied novel linked-read genome sequencing technology to identify novel causes of MGORS, a condition normally inherited in an autosomal recessive manner. We identified biallelic variants in four individuals in *DONSON*. All variants were proven to be deleterious; missense variants altered the subcellular localisation of DONSON, while a deep intronic variant created a novel splice acceptor site, changing the reading frame of the transcript.

Our identification of four individuals with variants in *DONSON* establishes it as a *bona fide* MGORS disease gene. While extensive clinical information is not available for all subjects in this DONSON-MGORS cohort, these individuals were clinically diagnosed as MGORS prior to genetic analysis, using features of reduced growth, microtia and patellar a/hypoplasia. Combining this clinical assessment of these individuals alongside the known cellular role of DONSON further reiterates that MGORS is a disorder of DNA replication. In total, nine genes are now associated with MGORS. All have separate but linked roles in DNA replication, which confirms that the cardinal features of MGORS—proportionate growth reduction, microtia and patellar hypo/aplasia—are all caused by disruption to DNA replication. While the growth retardation is commonly observed in genetic disruption of proliferation genes, the pathophysiological mechanism causing the microtia and patellar a/hypoplasia remains unclear.

The currently known MGORS genes encode members of the preRC and preIC complex, which act at various steps prior to active DNA replication.^27^ Functional characterisation of DONSON places the protein as a member of the replisome at the replication fork where it acts as a surveillance factor for DNA damage.^20^ This is further downstream in the DNA replication process compared with other MGORS-associated genes and presents an intriguing hypothesis—could DONSON have additional roles in replication upstream to what has presently been characterised? Such a role would be in alignment with the specific phenotypic features associated with MGORS, particularly the microtia and patella anomalies, and the strong facial gestalt of P4. Alternatively, identifying *DONSON* as an MGORS gene, given its role at the replication fork, could suggest the mechanistic basis of MGORS is broader than previously thought. Recently, pathogenic variants in *POLE*, encoding the catalytic subunit of polymerase ε, were associated with IMAGe syndrome (MIM: 600856).^28^ In addition to the primordial dwarfism, several subjects also had absent patella and many had microtia as part of a wider collection of clinical features. While such findings could point to MGORS (or ‘MGORS-like’) being a general disorder of DNA replication initiation, the non-growth phenotypes caused by pathogenic variants in other preRC/preIC components including *MCM4* and *GINS1*, do not overlap, indicating the biology is complex.

With these new findings, we have three clinically distinct phenotypes all linked to hypomorphic variants in the same gene. While technically these disorders should be considered allelic, the reasons for such a spectrum of growth restriction and skeletal disruption is not clear. The severity of the impact imposed by the individual missense variants on nuclear DONSON levels could point towards a genotype–phenotype correlation; however, there is insufficient quantitative evidence for each variant to conclude this—further comparison of variants on DONSON function would provide valuable insight. Furthermore, the clinical differences do not immediately suggest a straightforward spectrum of severity. The relationship between head size and height is not linearly correlated across all three cohorts; a defining feature of MIMIS individuals is the micromelia reducing limb length rather than an overall reduced stature incorporating the torso.^23 24^ Similarly, the differences in skeletal anomalies is striking. Mild skeletal features in MISSLA individuals include thumb dysplasia, clinically reminiscent of Fancomi anaemia,^20 22^ whereas severe examples in MIMIS cases include radial/ulna synostosis, absent radii and missing or hypoplastic digits in both hands and feet.^24^ The skeletal anomalies associated with MGORS have not been surveyed in detail,^7^ and except for several reports of retroflexed knees, no other features are usually clinically overt.^6^ Microcephaly in MIMIS and MISSLA cohorts is of similar severity, while in MGORS, it is milder, as illustrated by the head size of P3 being reported well within the normal range.

This study provides one of the first examples of using Chromium linked-read technology for variant phasing to identify a novel disease gene. Standard DNA samples obtained through clinical laboratories were successfully used, illustrating that a specific DNA extraction protocol is not required for the generation of high molecular weight DNA. Using traditional trio sequencing approaches, parents are sequenced to determine biallelic variants segregating *in trans* rather than *in cis* in the patient, which serves as a very effective filtering step. The use of Chromium linked-read sequencing relieves the requirement of sequencing parents in order to identify candidate genes with biallelic variants, providing a cost-effective solution and serves particular benefit when one or both parental samples are not available. MGORS is predominantly an autosomal recessive disorder (*GMNN* variants are the sole exception to date^11^) and therefore under this hypothesis, Chromium genome sequencing was an appropriate technology to use to ascertain candidate disease genes and variants. In considering the broader utility of this technology for gene identification, sequencing of parental samples would still be required for *de novo* variant identification.
